# Beyond the Barrier: Epithelial Cells as Immune Sentinels in the Female Genital Tract

**DOI:** 10.1111/aji.70243

**Published:** 2026-05-06

**Authors:** Lauren Jirik, Shreya Joshi, Aisha Nazli, Charu Kaushic

**Affiliations:** ^1^ McMaster Immunology Research Centre, Michael G. DeGroote Centre For Learning and Discovery McMaster University Hamilton Ontario Canada; ^2^ Department of Medicine McMaster University Hamilton Ontario Canada

**Keywords:** epithelial cells, female genital tract, HIV‐1, HSV‐2, immune responses, sexually transmitted infections

## Abstract

Epithelial cells (ECs) of the female genital tract (FGT) serve as an essential barrier and the first line of defense against sexually transmitted pathogens. Beyond providing a physical barrier, these cells actively contribute to immune responses through pathogen recognition, cytokine release, and modulation of adaptive immune responses. Sexually transmitted viruses such as HIV‐1 and HSV‐2 must overcome the physical and functional barriers of the mucosal surface to establish infection. This review explores the intricate relationship between genital ECs and HIV‐1 and HSV‐2, emphasizing on how these interactions influence infection outcomes. We examine the innate immune responses of ECs in the upper and lower FGT, highlighting both their similarities and differences. Additionally, we delve into the mechanisms of pathogen recognition and virus‐specific innate immune responses of genital ECs to HIV‐1 and HSV‐2. Deepening our understanding of epithelial‐viral interactions is critical for identifying key determinants of susceptibility and resistance to sexually transmitted infections (STIs). Elucidating these mechanisms is essential for developing targeted strategies to enhance mucosal immunity, through novel antiviral therapies, vaccine strategies, or interventions to fortify epithelial defenses. Such advancements have the potential to improve protection against these infections and reduce their global burden.

## 1 Introduction

Mucosal epithelial cells (ECs) play a central role in providing the primary barrier between the body and the environment outside, while also initiating and orchestrating immune responses in the mucosal tissues [1]. Their functions include secreting various antiviral proteins, recognizing pathogen‐specific structures, and producing antimicrobial mediators, such as epithelial defensins, both constitutively and in response to infection [1]. Furthermore, ECs release a diverse array of cytokines and chemokines that recruit leukocytes to the site of infection and influence the activation of the adaptive immune response. The ECs of the female genital tract (FGT) maintain mucosal barrier integrity and act as the first line of defense against sexually transmitted pathogens. The FGT is divided into two distinct anatomical regions: the lower and upper genital tract. The lower genital tract consists of the vagina and ectocervix, where initial exposure to viral pathogens typically occurs during vaginal intercourse with an infected male partner. Exposure to these pathogens can also occur in the upper genital tract, which includes the endocervix, uterus, fallopian tubes, and ovaries. The FGT is a unique immunological site which must protect the mucosa from a variety of opportunistic pathogens without compromising key reproductive functions such as sperm and ovum transport for fertilization, implantation, and fetal support throughout gestation. The FGT is highly sensitive to sex steroid hormones, estrogen and progesterone, which fluctuate cyclically during the menstrual cycle. These hormones regulate the immune system throughout the FGT to optimize conditions for successful reproduction and to protect against pathogens. As a result, they influence susceptibility to sexually transmitted infections (STIs) through both direct and indirect mechanisms.

Human Immunodeficiency Virus (HIV‐1) and Herpes Simplex Virus type 2 (HSV‐2) are significant global health challenges, particularly for women. In 2024, approximately 40.8 million people worldwide were living with HIV‐1, with women and girls accounting for 53% of these cases [2]. Transmission through the FGT is five times more likely to occur than through the male genital tract and accounts for 40% of annual HIV transmissions [3]. Heterosexual transmission via the FGT is the primary route of acquiring infection for women, highlighting the critical need to understand host‐pathogen interactions within the FGT to improve HIV‐1 prevention strategies [3]. HSV‐2, one of the most common STIs globally, affects 519.5 million individuals aged 15–49, with 25.6 million new cases being recorded annually as of 2020 [4, 5]. Similar to HIV‐1, women are disproportionately affected, being infected twice as often as men [6]. HSV‐2 predominantly targets the vaginal mucosal epithelium, making it the leading cause of recurrent genital lesions in women, exacerbating mucosal vulnerability to HIV‐1, and occasionally resulting in severe complications such as encephalitis or ocular infections [4, 5, 7]. Antiviral therapies exist for both HIV‐1 and HSV‐2, offering effective viral suppression and symptom control, but they do not eliminate the infections. Neither virus currently has an approved vaccine. Together, these infections underscore the urgent need to explore the interactions between sexually transmitted viruses and genital ECs to develop more effective prevention and treatment strategies tailored to women's health.

This review will detail the innate immune functions of ECs within the FGT, emphasizing their crucial role in establishing an immediate defense and modulating adaptive immune responses to provide comprehensive protection against HIV‐1 and HSV‐2.

## 2 Physical Barriers Within the Female Reproductive Tract

**FIGURE 1:**
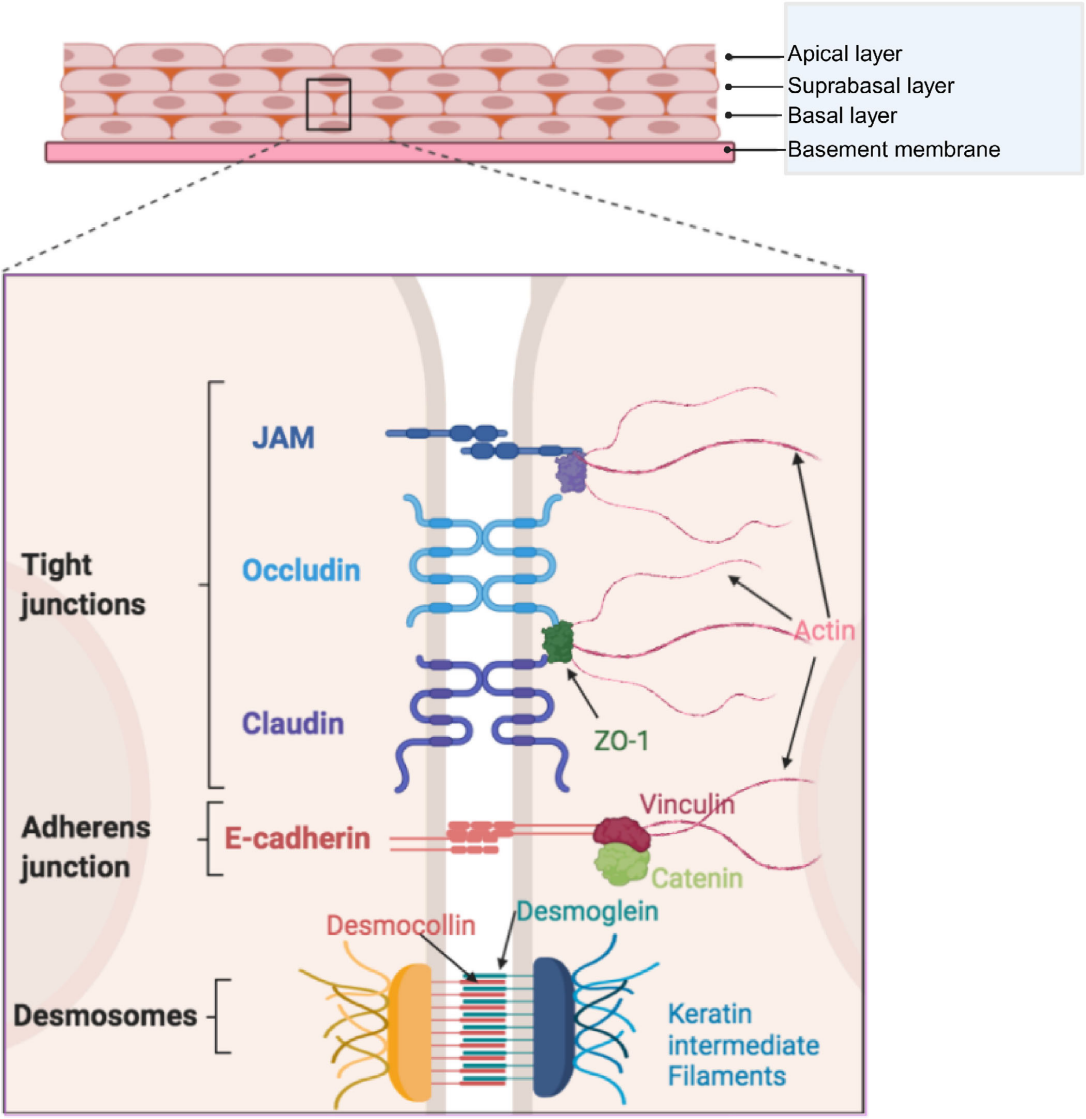
A summary of cell‐cell adhesion proteins in the vaginal epithelium. The three main types of cell‐cell adhesion complexes are tight junctions, adherens junctions, and desmosomes. Tight junctions are composed of transmembrane proteins including claudins, occludin, and junctional adhesion molecules (JAM). Zona Occludens (ZOs) like ZO‐1 attach to transmembrane proteins like claudins and occludin and regulate their architecture and function. Adherens junctions are made up of epithelial cadherin (E‐cadherin) dimers and are attached to actin filaments through catenin and molecules that interact with actin like vinculin. Desmoglein and desmocollin are desmosomal adhesion molecules that attach to the keratin intermediate filaments of the cytoskeleton through an intracellular anchor. This image was created using BioRender.com.

The lower FGT is lined by stratified squamous ECs, which constantly undergo shedding, renewal and differentiation, creating a multilayered protective barrier [8]. This epithelium contains several distinct layers including a mitotically active basal layer, the suprabasal layer, and a superficial layer of cornified cells, all of which rest upon a dense layer of stromal fibroblasts which form the lamina propria [9, 10, 11]. This multilayered epithelium acts as a mechanical barrier, reducing the risk of viral infection by shielding underlying immune cells located within the lamina propria that are targets for HIV‐1. Moreover, the constant shedding of superficial cells prevents pathogen entry and colonization [12]. Intercellular junction complexes, such as tight junctions, adherens junctions, and desmosomes maintain epithelial organization and barrier integrity, inhibiting paracellular viral entry [13, 14, 15]. Tight junctions consist of transmembrane proteins that form a seal across the intercellular space, such as zonula occludens (ZOs), claudins, and junctional adhesion molecules (JAM), as shown in Figure 1, which restrict the paracellular passage of pathogens across the epithelium [15, 16]. Below tight junctions, adherens junctions connect actin filaments between cells to form a continuous adhesion belt, such as E‐cadherin (Figure 1), while desmosomes link keratin intermediate filaments, creating a strong structural framework [14, 17]. The most abundant junctional proteins within the ectocervix and vagina are ZO‐1, E‐cadherin, JAM‐A, JAM3, and claudin‐1 [13]. Notably, the most apical layers of the vaginal and ectocervical epithelium lack these junctions, resulting in a “leaky” superficial layer increasing potential entry points for viral pathogens [18]. An additional region of increased susceptibility is the squamo‐columnar junction between the ectocervix and the endocervix, where the squamous epithelium changes to the single layer of columnar epithelium, referred to as the transformation zone. Beyond this morphological shift, the transformation zone is the most immunologically active site in the reproductive tract. Within healthy women, this region contains a dense population of lymphocytes and antigen presenting cells, which increase further during inflammatory conditions [19]. The combination of epithelial remodeling, relative structural fragility, and an enriched target population could easily render this site particularly permissive to viral entry. Indeed, clinical studies have shown that cervical ectopy, where the columnar ECs of the endocervix protrude into the ectocervix and are exposed to the vaginal milieu, is associated with an increased risk of heterosexual transmission of HIV‐1 [20].

In addition to providing a physical barrier, ECs secrete mucins into the vaginal lumen, forming a hydrophobic, viscoelastic gel composed primarily of large mucin glycoproteins [21]. These high–molecular weight, heavily glycosylated proteins are central components of innate immunity and exist as either membrane‐bound mucins (including MUC1, MUC4, MUC13, and MUC16) or secreted gel‐forming mucins (such as MUC5AC, MUC5B, and MUC6). Ectocervical and vaginal ECs express MUC1 and MUC4, with expression of MUC4 being uneven throughout the vagina [22]. This mucus layer acts as a lubricant and provides an added layer of physical defense by restricting motility of pathogens, limiting their access to underlying ECs [23, 24]. The carbohydrate groups within mucin glycoproteins can serve as binding sites for viruses and bacterial membrane receptors, inhibiting attachment to the EC surface [21]. As reviewed in [25] crude saliva and its purified mucins (MUC5B and MUC7), as well as the purified mucins from breast milk (MUC1 and MUC4), and pregnancy plug cervical mucus (MUC2, MUC5AC, MUC5B and MUC6), have been shown to inhibit HIV‐1 in vitro. Moreover, mucus serves as a medium for various host defense molecules, including immunoglobulins (Ig), antimicrobial peptides (AMPs), antibacterial enzymes, and components of the complement system, which can directly neutralize pathogens [21]. In the FGT, cervicovaginal mucus treated with HSV‐1‐binging IgG could trap the virus and IgG trapping potency was correlated with the menstrual cycle [26, 27, 28]. Sex hormones play an essential role in modulating the quantity and composition of the vaginal mucus. For instance, high levels of estradiol during ovulation produces mucus with a low viscosity, enabling efficient movement of sperm [29, 30]. On the contrary, increased levels of progesterone during the luteal phase results in a thick, viscous mucus which thwarts the passage of substances from the lower FGT to the upper FGT [29, 30]. This thickness in mucus can be associated with a peak in MUC4 expression during the luteal phase [31]. Furthermore, estrogen has been shown to stimulate expression of the MUC1 gene in mice, while progesterone inhibits this activation [26]. One of the major alterations observed during the menstrual cycle was within the O‐glycosylation of the mucins, specifically during ovulation [32]. Andersch‐Björkman et al. [32] found an increase in the relative abundance of neutral oligosaccharides versus acidic ones during ovulation. The characteristic changes observed at ovulation included a reduction in NeuAcα2–6 linked to GalNAcol and NeuAcα2–3 linked to Gal–GalNAcol, accompanied by an increase in Core 2 glycans (Galβ1–3(GlcNAcβ1–6)GalNAcol), which are typically fucosylated [32]. These alterations likely reflect enhanced Core 2 β1,6‐N‐acetylglucosaminyltransferase activity together with reduced sialyltransferase activity [32].

In addition to these protective mechanisms, the vaginal mucosa is colonized by an indigenous microbial community which exists in a symbiotic relationship with the host, capable of influencing the physiology and immune function of the FGT [33]. An optimal vaginal microbiota is characterized by low‐diversity and high abundance of *Lactobacillus* species [33, 34, 35, 36]. *Lactobacilli* bolster the immune system by providing broad, non‐specific defense against an array of pathogens. This is achieved through the secretion of anti‐microbial bacteriocins, the formation of biofilms, and maintaining low pH levels via lactic acid and hydrogen peroxide production [34, 37]. This protective environment is supported through the production of glycogen by ECs, which can be released into the vaginal lumen, where it acts as critical nutrient source for *Lactobacillus* species [38]*. Lactobacillus crispatus* in the vaginal microbiota has been correlated with protection against STIs and various adverse reproductive outcomes. Disruption of the microbiota, by increased abundance of anaerobes such as *Gardnerella vaginalis* and *Prevotella bivia*, results in reduced viability of vaginal ECs and compromised epithelial barrier integrity through the production of inflammatory cytokines, resulting in the disruption of tight junction proteins such as ZO‐1 and occludin [39, 40]. This predisposes women to adverse reproductive outcomes and increases susceptibility to STIs [36, 41]. Data from our has shown that presence of *L. crispatus* has been shown to negate the adverse effects caused these dysbiosis associated species when cocultured with these species and vaginal ECs. We have found that *L. crispatus* produces hydrogen peroxide, and its supernatant can inhibit the growth of *G. vaginalis* and *P. bivia* [40].

Periods of significant hormonal change, such as puberty and menopause, coincide with pronounced shifts in the vaginal microbiome (VMB) [42, 43, 44, 45]. The rise in estrogen during puberty is associated with a transition from predominantly diverse anaerobic communities to microbiota enriched in *Lactobacillus* species. In contrast, the decline in estrogen that accompanies menopause is linked to reduced *Lactobacillus* dominance and greater microbial diversity, with postmenopausal women significantly more likely to harbor heterogeneous bacterial communities [46, 47]. The observation that estradiol‐based hormone replacement therapy promotes restoration of *Lactobacillus* predominance further supports a hormone‐dependent effect on microbial composition [47, 48].

In contrast to the lower tract, the upper FGT is lined by a single layer of columnar ECs joined by tight junctions to provide physical protection against opportunistic and pathogenic microbes [49]. Endocervical epithelium express MUC1, MUC4, MUC5AC, MUC5B, and small amounts of MUC6, to create a protective mucus layer over the columnar cells [22]. The only mucin detected within the fallopian tube was MUC1. Recent studies report a stable presence of the vagino‐uterine microbiome, although the quantity of bacteria within the upper FGT is approximately 2–4 log orders lower than the vaginal tract [50, 51, 52]. ECs in the endocervix are highly organized, with specialized junctional complexes that restrict microbial passage. The apical side of endocervical ECs are joined by juxtaluminal tight junctions, while neighboring cell membranes are fused to effectively seal the apex of each cell, preventing intercellular passage of luminal contents [13]. Expression of JAM‐A, E‐cadherin, ZO‐1, claudin‐1, and occludin is found within the simple columnar epithelium of the endocervix [13, 53, 54]. This structural organization fortifies the barrier against pathogens and positions ECs as critical sensors of their environment.

## 3 Epithelial Cell Role in Sensing the Environment in FGT

Upon pathogen contact with epithelial surfaces, signaling cascades are activated, resulting in the production of chemokines, cytokines, prostaglandins, and leukotrienes by ECs [55]. These responses are primarily driven by the detection of pathogen‐associated molecular patterns (PAMPs) and endogenous damage‐associated molecular patterns (DAMPs) via specific pattern recognition receptors (PRRs) [56]. PAMPs are highly conserved microbial structures, including lipopolysaccharide (LPS), lipoproteins, peptidoglycan (PGN), lipoarabinomannan, and oligosaccharides [57]. DAMPs are released by dying, injured, or stressed cells which may occur during exposure or infection with a pathogen. Common DAMPs include ATP, heat‐shock proteins, and HMGB1. Key PRR families include Toll‐like receptors (TLRs), nucleotide‐binding oligomerization domain (NOD)‐like receptors, retinoic acid‐inducible gene I (RIG‐I)‐like receptors, formyl peptide receptors, mannose and glycan receptors, C‐type lectin receptors, complement receptors, and CD14 [58, 59]. Additionally, genital ECs are equipped with TLR‐independent DNA sensors, such as RNA polymerase III and IFI16 which regulate type I interferon (IFN) induction through activation of the transcription factor IRF3 or the cyclic GMP‐AMP synthase (cGAS)‐stimulator of IFN genes (STING) pathway [60, 61, 62, 63].

TLRs sense a diverse array of pathogens, including bacteria, fungi, protozoa, and viruses. To date, at least 10 human TLRs and 13 mouse TLRs have been described [64]. TLR1, 2, 4, 5, and 6 are located on the plasma membrane of ECs and detect pathogen membrane components, while TLR 3, 7, 8, and 9 are expressed in cytoplasmic organelles, mainly within endosomes, lysosomes, endolysosomes, and endoplasmic reticulum where they detect pathogen‐derived nucleic acids [57, 64]. TLR2 in concert with TLR1 or TLR6 discriminates between the molecular patterns of triacyl and diacyl lipopeptide, respectively. TLR4 recognizes bacterial lipopolysaccharides (LPS), while TLR5 recognizes bacterial flagellin [65, 66, 67]. It is important to note that recognition of PAMPs by TLRs 1,2,4,5, and 6 are not confined only to bacterial components, as they can also recognize various classes of pathogens. Nucleic acids are recognized by TLR 3, 7, 8, and 9, with TLR 7 and 8 recognizing single stranded RNA, while TLR9 recognizes DNA containing unmethylated cytosine‐phosphate‐guanine (CpG) motifs, which are found in bacteria and viruses, and TLR3 recognizing double‐stranded RNA (dsRNA) [68, 69, 70, 71, 72].

**FIGURE 2:**
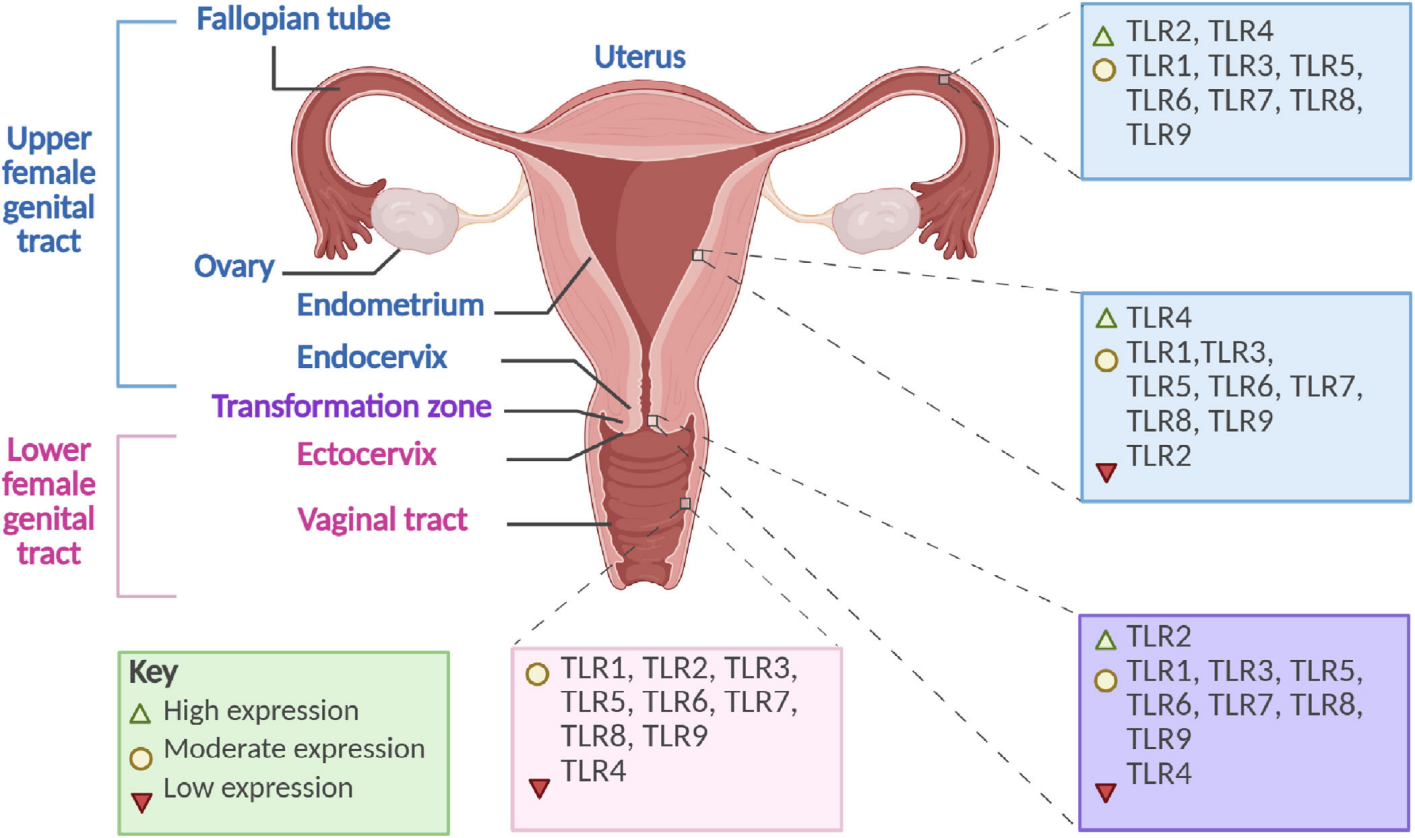
The expression of Toll‐like receptors (TLRs) throughout the female genital tract. An illustration depicting the anatomy of the female genital tract (FGT) and TLR expression levels and localization. Several studies have detected expression of TLR1, TLR3, TLR5, TLR6, TLR7, TLR8, and TLR9 in the fallopian tubes, endometrium, cervix, and vagina. TLR2 is also expressed in these regions, with the highest mRNA levels observed in the fallopian tubes and cervix, and markedly lower expression in the endometrium. The expression of TLR4 remains a subject of debate, with some studies reporting its presence in the epithelial cells of the fallopian tubes, endometrium, endocervix, and vagina, while others indicate its absence in the fallopian tubes, endocervix, ectocervix, and vagina. Despite these discrepancies, evidence suggests a progressive decline in TLR4 expression along the FGT, with the highest levels detected in the fallopian tubes and endometrium. Created with BioRender.com.

Expression of TLR1, TLR3, TLR5, TLR6, TLR7, TLR8, and TLR9 has been observed across the human FGT, including in the fallopian tubes, endometrium, cervix, and vagina (Table 1) [73, 74, 75, 76, 77, 78, 79]. TLR2 expression has been reported in the fallopian tubes, endometrium, cervix, and vagina, with the highest mRNA levels in the fallopian tubes and cervix, with significantly reduced expression in the endometrium [73]. Notably, TLR2, TLR3, TLR5, and TLR6 exhibit significantly elevated expression in the endometrium during the secretory phase of the menstrual cycle [80]. Conflicting findings have emerged regarding TLR4 expression in FGT epithelia. Some studies report TLR4 presence in the ECs of the fallopian tubes, endometrium, endocervix, and vagina while others report its absence in the ECs of the fallopian tubes, endocervix, ectocervix, and vagina [76, 81, 82]. Despite these discrepancies, the overall expression of TLR4 appears to decline progressively along the tract, with the highest levels of expression being found in the fallopian tubes and endometrium [73]. A summary of the expression and location of these TLRs within the FGT is presented in Figure 2 and Table 1.

**TABLE 1:** A summary of various innate immune molecules produced by epithelial cells of lower and upper FGT.

FGT Region	Tight junctions and adhesion molecules	Mucins	TLRs	Cytokines	Chemokines	AMPs
**Lower** (Vagina and ectocervix)	ZO‐1, E‐cadherin, JAM‐A, JAM3, and claudin‐1 (Apical layers lack these junction proteins) [13]	*MUC1*, *MUC4* [22]	TLR1, TLR2, TLR3, TLR5, TLR6, TLR7, TLR8, and TLR9 [74, 75] TLR4 (Low expression) [73]	TNF‐α, IL‐6 [82, 83]	MIP‐1α, MIP‐1β, IL‐8 [82, 83]	HD5 (Vagina) [84] HBD‐1, 2 [77] LL‐37/hCAP‐18 [85] SLPI [85] Trappin‐2/Elafin [85, 86, 87] Lysozyme [88, 89] Psoriasin (S100A7) [90] SPA [91] SPD [92] Lactoferrin [93]
**Upper** (Endocervix, uterus, fallopian tubes, and ovaries)	JAM‐A, E‐cadherin, ZO‐1, and occludin (Endocervical cells) [82, 83]	*MUC1*, *MUC6* (Endometrial cells) [22]	TLR1, TLR3, TLR5, TLR6, TLR7, TLR8, and TLR9 [74, 78, 79] TLR2 (Highest expression in fallopian tubes and cervix, and low expression in endometrium) [73] TLR4 (Highest expression in fallopian tubes and endometrium) [73]	IL‐6, G‐CSF, CCL2, GM‐CSF, TNF‐ α [94] IL‐1β, IL‐6, IL‐10, IL‐18, CCL2, VEGF (High in cervix than endometrium) IL‐12, IL‐15, and MIF (Low in cervix than endometrium) [95]	MIP‐1β and IL‐8 [94]	HD5 (Endometrium, cervix) [84, 96] HBD‐1, 2 [77, 78] LL‐37/hCAP‐18 [85, 97] SLPI [59, 85, 87] Trappin‐2/Elafin [59, 85] HE4 [98] Lysozyme [88, 89] S100P (Endometrium) [99] SPD [92] Lactoferrin [93]

Upon activation of the TLR pathway, transduction is mediated by the recruitment of two intra‐cellular signaling cascades: MyD88‐dependent cascade leading to secretion of pro‐inflammatory cytokines or TRIF‐dependent cascade resulting in the induction of type 1 IFNs as well as inflammatory cytokines and chemokines [55, 65]. Exposure of uterine ECs to the TLR3 agonist polyinosinic:polycytidylic acid (poly(I:C)), results in the production of pro‐inflammatory cytokines, including tumor necrosis factor alpha (TNF‐α), interleukin 6 (IL‐6), granulocyte‐macrophage colony‐stimulating factor (GM‐CSF), and granulocyte colony stimulating factor (G‐CSF), as well as the chemokines: interleukin 8 (IL‐8,)  monocyte chemoattractant protein‐1 (MCP‐1), and macrophage inflammatory protein‐1 beta (MIP‐1b) [69]. This exposure also initiates a strong antiviral response, evidenced by the induction of IFN‐β, myxovirus resistance gene 1 (MX1), and 2’‐5’ oligoadenylate synthetase (OAS) mRNA expression [59]. Activation of TLR3 in ectocervical and vaginal ECs results in increased production of IL‐6, GM‐CSF, as well as IL‐8, IL‐2R, IFN‐α, and IL‐7 [100]. Stimulation of TLR7/8 results in the elevated production of pro‐inflammatory markers such as IL‐6 and GM‐CSF across all compartments of the FGT [100]. In the endocervix, the overexpression of these cytokines is associated with an increased expression of lymphocyte‐attracting chemokines such as CCL2, CXCL9, CCL3, CCL4, CCL5, and IL‐10 [100]. When TLR4 is stimulated, an upregulation of GM‐CSF, CCL2, IL‐2R, CCL3, and IL‐7 expression is observed in both the uterus and vagina, with a pronounced effect seen in the cervix [100]. In the ectocervix, TLR4 activation results in overexpression of IL‐8, IL‐1RA, and IFN‐α in the ectocervix, while CCL4 is elevated in the endocervix [100]. TLR9 activation enhances IL‐8 secretion across all compartments of the FRT except the endocervix [100]. Specifically, within the vagina and endocervix, IL‐6 production is increased, while in the ectocervix, TLR9 activation stimulates increased secretion of IL‐2R, IFN‐α, and IL‐7, while overexpression of IL‐2R, GM‐CSF, CCL4, and IL‐12 occurs in the endocervix [100]. Other PRRs, such as NOD‐like receptors, play a crucial role in pathogen sensing within the FGT [58]. NODs are cytoplasmic PRRs that recognize muropeptides derived from the degradation of PGN from bacterial cell walls [101]. The receptor‐interacting serine‐threonine kinase 1 (RIPK2) plays a crucial role in the cell signaling of both NOD1 and NOD2, triggering signaling of the transcription factor nuclear factor kappa B (NFκB), leading to the production of pro‐inflammatory cytokines and chemokines, including CXCL8 [101, 102, 103]. NOD1 and NOD2 receptors are distributed throughout the FGT, with a notable concentration in the endometrium [59]. NOD1 is constitutively expressed, while NOD2 expression increases during the late secretory phase of the menstrual cycle and is selectively upregulated during HIV‐1 infection, a response that can limit viral replication [59, 104]. Retinoic acid‐inducible gene I (RIG‐I) and melanoma differentiation‐associated gene 5 (MDA5) are key members of the RIG‐I‐like receptor (RLR) family. These sensors detect viral RNA and initiate a classical antiviral response, utilizing the mitochondrial adaptor protein (MAVS), resulting in the production of inflammatory cytokines and type I IFNs, which ultimately inhibit viral replication and transmission [105]. Additionally, RLRs have been shown to recognize poly(I:C), a synthetic analog of double‐stranded RNA [106]. ECs from all compartments of the FGT constitutively express RIG‐1 and MDA5 receptors, with the highest expression in the fallopian tubes [107]. Activation of these receptors leads to the phosphorylation of transcription factors IRF3 and IRF7 by TANK‐binding kinase 1 (TBK1) and inhibitor of NFκB kinase‐ε (IKKε), triggering the expression of type I IFNs and inflammatory cytokines that assist in viral clearance [108].

## 4 Epithelial Cell Mediated Innate Immune Responses in the FGT

The rapid detection of pathogens by PRRs expressed by ECs initiates the overall innate immune cascade involving activation of signaling pathways that lead to production of pro‐inflammatory cytokines and chemokines [109]. However, owing to the differences in anatomical and immunological characteristics, as well as the influence of microbiota and sex hormones, the expression of these cytokines and chemokines differs greatly between the upper and lower FGT. A study by Kayisli et al. [110] showed that ECs of the cervix, endometrium and the fallopian tubes express seven cytokines and chemokines, IL‐8, IL‐6, G‐CSF, CCL2, GM‐CSF, TNFα, and MIP‐1β. All three tissues produce high levels of IL‐8 (neutrophil chemokine) which is associated with proliferation and angiogenesis in the early‐mid secretory phase of the menstrual cycle and apoptosis during menstruation [110]. Boomsma et al. [95] showed that the proteomic profile of cytokines and chemokines in the cervix and endometrium are distinct. Specifically, the levels of IL‐1β, IL‐6, IL‐10, IL‐18, CCL2, and vascular endothelial growth factor (VEGF) are significantly higher in cervical secretions compared to endometrial secretions, while IL‐12, IL‐15, and MIF are significantly lower [95]. Uterine ECs produce several chemokines including MIP‐1α, MIP‐1β, RANTES, and SDF‐1α, which prevent HIV‐1 from binding to host cell coreceptors‐ CCR5 and CXCR4 [111].

There are different challenges encountered by the immune system in the various compartments of the FRT, as a result of which there are also differences in immune responses between the lower (vaginal and ectocervix) and upper (uterus and endocervix) compartments [112]. *N. gonorrhoea* infection of primary endocervical ECs and immortalized End1/E6E7 cell line results in activation of TLR pathways triggering NFκB signaling to release pro‐inflammatory cytokines such as MIP‐1α, MIP‐1β, TNF‐α, IL‐6, IL‐8 [82]. In conditions like bacterial vaginosis (BV) where there is a predominance of anaerobes like *G. vaginalis* and *P. bivia*, an increased production of inflammatory cytokines including IL‐1α, IL‐1β and TNF‐α, TNF‐β, IL‐10, IL‐8, IL‐12, IL‐4, and a downregulation of chemokines such as CXCL10, CCL22 and MIP‐1α, IL‐7 and GM‐CSF [113, 114]. In contrast, studies have found that lactic acid produced by vaginal *Lactobacillus* decreases the production of TNF‐α, IL‐6, and IL‐8 by vaginal ECs leading to an anti‐inflammatory response [115]. Wagner et al. [116] in their study on vaginal ECs demonstrated that estradiol suppressed the expression of mRNA encoding pro‐inflammatory cytokines TNF and IL‐1α. Thus implying that inflammation and subsequent immune cell influx to the EC surface is dampened prior to ovulation, the time which is most favorable for semen entry [116]. Throughout the menstrual cycle, estrogen controls the expression of IFN‐ɛ, a type I IFN, which is exclusively produced by the ECs of the FGT, but not IFN‐α and IFN‐β [117]. Furthermore, estrogen reduces ISG levels in response to type III IFNs in uterine ECs but has no effect on IFN‐ β (type I IFN) mediated ISG expression [117]. The fact that these cytokines and chemokines are constitutively secreted and are compartmentalized throughout the FGT emphasizes that ECs serve as gatekeepers of innate immune defense in the FGT.

## 5 Antimicrobial Defenses of FGT ECs

AMPs produced by ECs are at the forefront of defense against pathogens and are induced upon detection of pathogens. Located at the interface between the external environment and mucosal surface of the FGT, AMPs demonstrate not only direct antibacterial, antifungal and antiviral activities, but also pro‐/anti‐inflammatory activities, antiprotease activity, chemoattraction, apoptosis, and cell differentiation [118]. Owing to the continuous exposure of the reproductive tract to external factors, and changes with hormonal fluctuations, AMPs are critical in maintaining a balanced microbial environment, contributing to reproductive health.

The activation of PRRs via PAMPs triggers the downstream activation of transcription factors that in turn induce the transcription of AMPs [118]. Defensins, cathelicidins, lysozyme, S100 proteins, whey acid proteins, C‐type lectins, iron metabolism proteins, and kinocidins are the AMPs known to be expressed in FGT [118]. However, as reviewed by Wira et al. [119] and by Ghosh [120], some additional AMPs including bactericidal permeability‐increasing (BPI) protein, Thrombospondin‐1 (TSP‐1), histones, lipophilin, cystatin A, ubiquitin, and phospholipase A2 are also expressed by the FGT ECs. Defensins (reviewed in [121]), cathelicidins [122, 123], whey acid proteins (reviewed in [124]), lysozyme [109, 125, 126, 127], and iron metabolizing protein lactoferrin [128, 129, 130, 131] are broad spectrum AMPs having antibacterial, antifungal and antiviral (both anti‐HIV and anti‐HSV‐2) properties. S100 proteins on the other hand are antibacterial and antifungal in nature (reviewed in [118]), whereas C‐type lectins have antibacterial (reviewed in [118]) and antiviral activity, specifically, anti‐HIV [132].

Defensins are the most common type of AMPs found in humans [133]. They are a group of cationic, cysteine‐rich peptides, 18–45 amino acids long, with a central β‐sheet stabilized by three conserved intramolecular disulfide bonds. They are categorized into three groups, α, β, and θ defensins, based on the position of their disulfide bonds [134]. In α‐defensins, the alignment of the disulfide bonds is 1–6, 2–4, and 3–5 whereas in β‐defensins, the alignment format is 1–5, 2–4, and 3–6 [135].

Human Defensin 5 (HD5), a type of α‐defensin, is expressed throughout the FGT, by endometrial, cervical and vaginal ECs [84, 96]. Wu et al. [136] showed that endo‐ and ecto‐cervical ECs express HD6 mRNA, however, the mature HD6 has not been found in the upper nor the lower FGT. Human β‐defensin 1 (HBD‐1) is constitutively expressed by both upper and lower FGT ECs, but its levels are increased upon exposure to specific immune‐stimulating molecules such as TLR‐3 agonists [77]. Several studies have shown that HBD2 is expressed throughout the FGT [77, 85, 86, 137, 138]. Additionally, β‐defensins, specifically HBD3, enhances the transepithelial resistance, thus maintaining epithelial barrier integrity [139]. Han et al. [140] found that progesterone inhibited expression of HBD‐2 under the influence of LPS. Estradiol on the other hand, had no effect on HBD‐2. In the endometrium, the defensin HBD4 is upregulated by estrogen during the proliferative phase [141] whereas HBD2 is found to be highest during menses [142]. θ‐defensins, also known as mini defensins, are circular AMPs that are highly effective against STIs including HIV‐1, HSV‐2, and BV associated bacteria *G. vaginalis*. Retrocyclin, a class of θ‐defensin was shown to inhibit the cytolytic activity of *G. vaginalis* toxin, vaginolysin, along with negatively affecting biofilm formation. LL‐37/hCAP‐18, a type of positively charged Cathelicidin, another class of AMP, attaches to the negatively charged phospholipids on the surface of microbial cell membranes and exerts its antimicrobial effects [143]. Studies have shown that in the FGT, LL‐37 is expressed by the ECs of the endometrium, cervix and vagina [85, 97].

The Whey acid proteins (WAPs) are essentially protease inhibitors, with many of them possessing antimicrobial and immunomodulatory activity [144]. Secretory Leukocyte Protease Inhibitor (SLPI), Trappin‐2/Elafin, and Human Epididymis protein 4 (HE4) are the three WAPs expressed in the FGT. SLPI is expressed by both upper and lower FGT [85, 86, 87]. In addition to acting as protease inhibitor, SLPI also inhibits NFκB mediated inflammation, maintains homeostasis at barrier sites such as FGT, and prevents tissue destruction [124]. SLPI level is regulated by estradiol and is significantly reduced after menopause (reviewed in [120]). An important anti‐inflammatory mediator at the mucosal surfaces is the serine protease inhibitor, Trappin‐2/Elafin [145]. This AMP is constitutively produced by all ECs of the upper and lower FGT [138, 145, 146]. Ghosh et al. [120] found higher levels of Elafin in the cervico‐vaginal lavage of women in the secretory phase of the menstrual cycle than those in the proliferative phase, suggesting that its secretion is regulated by hormones within the FGT. Endometrial ECs were found to express more HE4 during the progesterone‐high secretory phase of the menstrual cycle [147].

Lysozyme, the first reported human‐derived AMP, is expressed by cervical and vaginal ECs [88, 89]. The antibacterial effect of lysozyme comes from its ability to break the PGN of the bacterial cell walls. It can directly interact with the cell membranes through its positively charged amino acids and is thus more effective against Gram positive bacteria and largely ineffective against Gram negative bacteria as they have a LPS containing outer membrane that prevents lysozyme from reaching the PGN layer [123, 148]. Expression of lysozyme is high during the estrogen high proliferative phase, drops mid‐cycle and rises again during the progesterone dominated secretory phase suggesting its hormone regulation (reviewed in [120]). S100 proteins, another class of AMPs are calcium binding proteins which are categorized into three subgroups based on their functions. Vaginal and ectocervical ECs constitutively express high levels of psoriasin (S100A7), a potent antibacterial protein, specifically targeting *E.coli* in vaginal fluid [90]. In the endometrium, the expression of another type of S100 protein, S100P is upregulated by progesterone, with no significant effect by estrogen [99]. The C‐type Lectins, Surfactant Protein A and D (SP‐A and SP‐D) are expressed by the ECs of the FGT. SP‐A is expressed by both the intermediate and the superficial layers of the vaginal epithelium [91]. SP‐D on the other hand, is expressed by ECs of the endometrium, cervix and vagina [92]. During the proliferative phase, estrogen elevates SP‐A levels, which falls during the secretory phase when progesterone rises. However, SP‐D is only expressed during the secretory phase of the menstrual cycle in conjunction with progesterone (reviewed in [120]). Uterine and vaginal ECs secrete lactoferrin into the cervicovaginal mucus to ensure lubrication and clearance of the microbes. Lactoferrins are highly suited for the acidic environment of the lower FGT, as their affinity towards iron increases at a lower pH [93]. The expression of lactoferrin in genital ECs is positively influenced by the circulating levels of estrogen, where increased expression is observed during the follicular phase and decreased in the luteal phase of the menstrual cycle [149]. Modulation of AMPs can provide a novel strategy to enhance protection in the FGT.

## 6 Viral‐Epithelial Cell Interactions and Innate Immune Responses During HIV‐1 Infection in FGT

While the genital epithelium itself is not infected by HIV‐1, it serves as a critical initial barrier to viral entry as well as recognizing the virus and initiating a cascade of innate immune responses that influence the outcome of exposure. In this section, we examine the mechanisms of HIV‐1 entry, its recognition by ECs, and innate immune responses generated by ECs against HIV‐1,

HIV‐1 employs multiple mechanisms to cross the genital epithelium, a critical step in initiating infection. The virus can cross the epithelial barrier through transcytosis, paracellular passage, or penetration via breaches in epithelial barrier [150, 151, 152, 153]. Viral entry is facilitated by expression of a number of receptors that can be utilized by the virus, including galactosylceramide (Gal‐Cer), C‐type lectins, and cell surface proteoglycans such as heparin sulphate, which can assist in viral adherence and penetration [154]. Studies suggest that gp340, expressed on cervical and vaginal ECs can facilitate HIV‐1 binding and translocation across the epithelial barrier [155]. While most studies have shown that HIV‐1 cannot replicate or become latent in ECs, HIV is recognized and internalized in these cells, leading to localized immune activation and viral persistence.

Primary uterine endometrial ECs recognize HIV‐1 mainly through interactions with its surface glycoprotein, gp120 [156, 157]. This interaction triggers the production of pro‐inflammatory cytokines such as TNF‐α, IL‐6, and IL‐8, which impair tight junctions and increase epithelial permeability, facilitating viral translocation [157]. TLRs, particularly TLR2 and TLR4, are central to this process [39]. Activation of these receptors by gp120 in the presence of heparan sulfate initiates signaling cascades that contribute to antiviral responses but also compromise epithelial barrier integrity [39]. This dual effect highlights the complexity of uterine EC responses to HIV‐1 and demonstrates their role in both defense and susceptibility. Different parts of HIV‐1 are recognized by other TLRs including TLR3, TLR7, and TLR8 expressed by ECs in the FGT (reviewed in [158]). Vaginal ECs actively participate in innate immunity by producing IFNs and AMPs in response to HIV‐1. IFN‐ɛ is expressed constitutively by vaginal ECs and has been found to induce restriction factors such as apolipoprotein B mRNA‐editing enzyme‐catalytic polypeptide‐like 3G (APOBEC3G), MX2, and TRIM5α, which inhibit viral replication [159]. IFN‐β is produced in response to gp120 via TLR2 signaling by upper genital ECs and has been shown to reinforce epithelial barrier integrity and reduce viral replication [160]. It does so by inducing the expression of APOBEC3G and inactive 2′,5′‐oligoadenylate synthetase (2′,5′‐OAS) which activates RNAse L (a latent endo‐ribonuclease) that degrades both viral and host RNAs, thereby disrupting protein synthesis [161]. Primary uterine, fallopian tube, cervical, and ectocervical ECs also secrete AMPs that inhibit HIV‐1 entry and replication. Trappin‐2/Elafin, effectively inhibits R5 and X4 tropic HIV‐1 strains [145, 162]. Human beta‐defensins, HBD‐2 and HBD‐3 produced by ECs of the genital mucosa, can disrupt the interaction between gp120 and CD4 receptor, effectively inhibiting infection, particularly X4 tropic strains [148, 163, 164]. Alternately, lactoferrin and SLPI block early stages of viral infection [154, 165, 166, 167]. The anti‐HIV activity of SLPI is limited only to HIV target cells including T cells, macrophages, and monocytes and there are two possible mechanisms of action: a) It prevents fusion of HIV with T cell plasma membrane by attaching to scramblase 1, a membrane protein that interacts with CD4 receptor and regulates the flow of the phospholipid bilayer of the plasma membrane [168]. b) In myeloid cells, SLPI blocks HIV entry by binding to annexin II, a macrophage receptor for phosphatidyl serine moiety that is picked up by budding virus from a productively infected cell, and stabilizes the fusion between the virus and neighboring cell, thus facilitating infection [144, 169]. In monocytes, SLPI acts right after HIV binding to host CD4 receptors and blocks viral DNA synthesis [170].

ECs of the FGT also secret mucus into the lumen which can trap HIV‐1 virions and restrict their motility [171] [23]. An in vitro study by Mall et al. [25] showed that several mucin proteins in the cervical mucus, (MUC2, MUC5AC, MUC5B and MUC6) have anti‐HIV activity.

**FIGURE 3:**
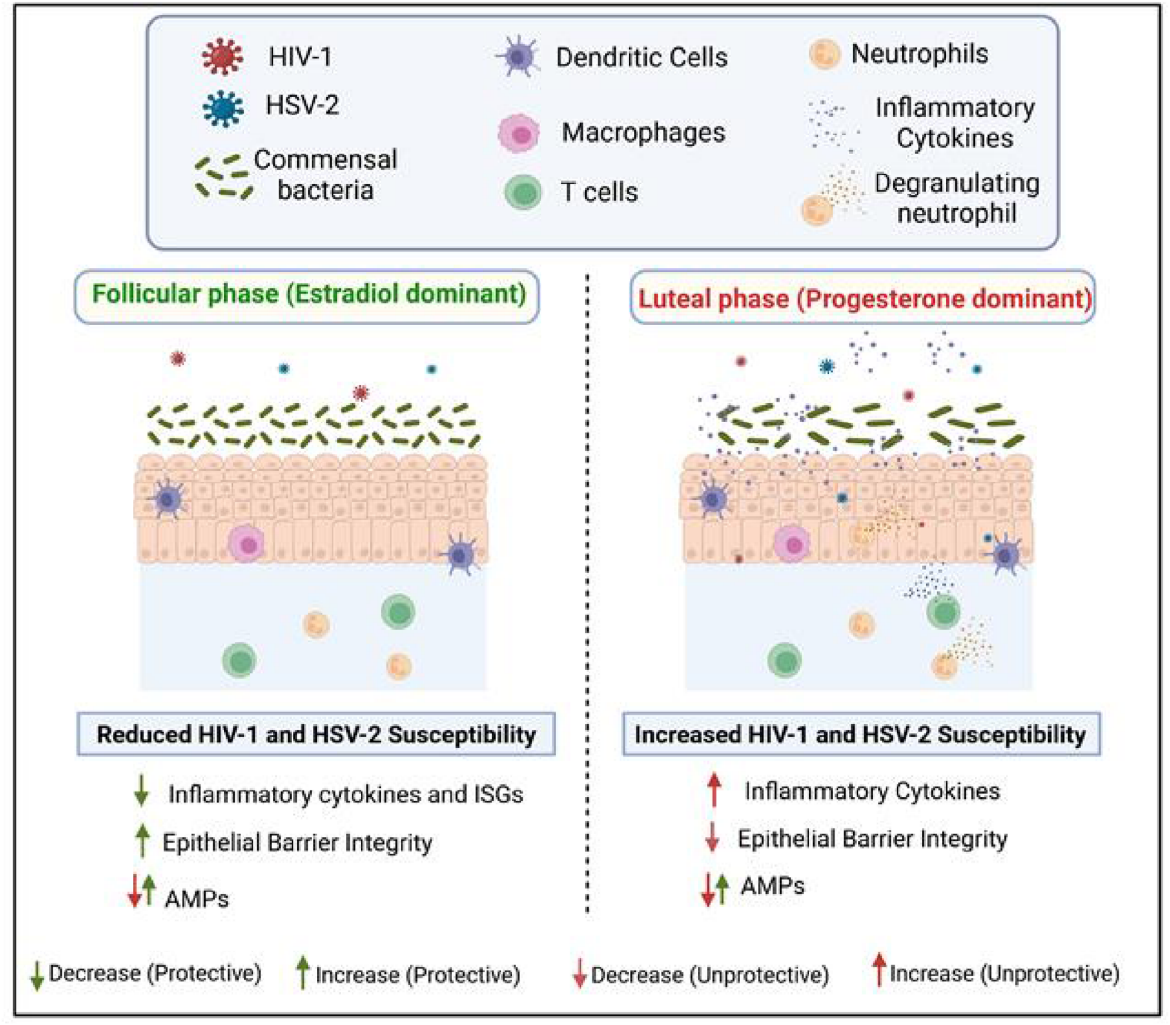
Hormonal influences on HIV‐1 and HSV‐2 susceptibility in FGT. A summary depicting impact of endogenous hormones on the FGT. The estrogen‐high follicular phase is characterized by an increase in junction proteins that lead to enhanced epithelial barrier integrity, reduced inflammation along with increase and decrease of certain AMPs all of which inhibit HIV‐1 and HSV‐2 binding and replication. The progesterone‐high luteal phase is characterized by a decrease in epithelial barrier integrity and an increase in inflammatory cytokines including TNF‐α, IL‐6, IL‐8 as well as modulation of AMP expression. These factors may collectively contribute to the increased HIV‐1 and HSV‐2 susceptibility in women observed during the luteal phase. Created with BioRender.com.

Given that cyclical changes in sex hormone levels impact immune responses in FGT, studies by Saba et al. showed that cervicovaginal tissue explants were able to sustain productive infection only if collected from women during their luteal phase indicating a “window of vulnerability” (about 7–10 days during the second phase of the menstrual cycle) to acquiring STIs during the progesterone‐high phase of menstrual cycle [172, 173]. Progesterone thus makes the vaginal microenvironment conducive, increasing the likelihood of acquiring STIs like HIV [174]. In an ex‐vivo study, primary endometrial ECs treated with hormones reported that estrogen enhanced barrier function as well as reduced pro‐inflammatory cytokines, TNF‐α, IL‐1α, and RANTES in response to HIV challenge [111]. A study from our lab has shown that primary endometrial ECs grown in estradiol are transcriptionally less responsive than those grown in progesterone, linking it to protection from HIV infection [175]. Collectively, these studies imply that progesterone creates an environment conducive for acquiring HIV infection in women whereas estradiol plays a protective role (Figure 3).

## 7 Viral‐Epithelial Cell Interactions and Innate Immune Responses During HSV‐2 Infection in FGT

While both HIV‐1 and HSV‐2 establish lifelong infections, their pathogenesis differs significantly. Unlike HIV‐1, which primarily targets CD4+ T cells in the FGT and establishes systemic infection, HSV‐2 directly infects and efficiently replicates in the ECs of the FGT causing localized mucosal damage and recurrent lesions. HSV‐2 is transmitted via contact with herpetic lesions, mucosal surfaces, or genital and oral secretions, preferentially infecting ECs [5, 176]. The viral entry process begins with attachment of the virus to the EC surface, which is mediated by multiple viral glycoproteins on the viral envelope and host cell receptors [177, 178]. This includes nectin‐1 and ‐2, herpesvirus entry mediator (HVEM), 3‐O sulfated heparan sulfate (3‐OHS), myelin‐associated glycoprotein (MAG), and paired immunoglobulin‐like receptor (PILRα) [179, 180, 181, 182, 183]. Nectin‐1 and nectin‐2 are the receptors most actively involved in HSV‐2 entry, especially in genital ECs where they are expressed throughout the menstrual cycle, with nectin‐1 serving as the primary receptor for viral entry [184, 185]. HSV‐2 infection of ECs in the FGT triggers various immune responses aimed at recognizing the virus and inhibiting viral spread. PRRs such as TLR2, binds to HSV glycoproteins gH/gL and gB, triggering the activation of NFκB signaling pathway [186]. ECs upregulate the expression of TLR2 and TLR4 on the surface of vaginal ECs, and initiate signaling pathways that enhance cellular immune defenses [76, 187, 188]. In primary vaginal ECs, upon recognition of HSV‐2, TLR2 translocates from the cell membrane to the Golgi complex, where it colocalizes with MyD88, an essential step in the TLR signaling cascade and cytokine production [189]. Furthermore, human cervical ECs upregulate expression of TLR4 and TLR9 following HSV‐2 infection, with TLR9 mRNA being upregulated as early as 2 hours post infection, with TLR9 being reported by several studies to recognize HSV‐2 DNA [190, 191, 192]. In primary cervical ECs viral replication induces the upregulation of intracellular TLR9 through the SP1/JNK signaling pathway, which is involved in type I IFN signaling and enhances the antiviral immune response [193]. TLR4 activation is associated with an inflammatory NFκB response in human cervical ECs and induces activator protein 1 (AP‐1) in all genital ECs [194, 195]. Additionally, both TLR9 and TLR2 activation drive NFκB‐driven transcription, further amplifying antiviral activity, through the NFκB dependent secretion of IL‐6 and IFN‐β [193]. Interestingly, when primary genital ECs are pretreated with poly(I:C), CpG A, and flagellin (ligands for TLRs), they show reduction in HSV‐2 replication due to upregulation of IRF3 and NFκB, and subsequently production of IFN‐β and nitric oxide [196]. Our lab has reported that HSV‐2 infection of vaginal ECs results in upregulation of TRIM26, a negative regulator of type I IFNs, which decreases nuclear localization of IRF3, thereby promoting viral replication [197]. In addition to TLRs, host DNA sensors such as cytosolic IFN‐gamma inducible 16 (IFI16) and DNA‐dependent activators of IFN (DAI) play a critical role in detecting HSV‐2 genetic material within infected primary vaginal ECs [60, 63, 189, 198]. These DNA sensors act as key components of the innate immune system, bridging early viral detection with a broader immune activation that limits viral replication. Infected genital ECs produce pro‐inflammatory cytokines, such as CCL2, IL‐8, IL‐6, IL‐22, and TNF‐α, which recruit immune cells to the site of infection [199, 200].

Although these inflammatory responses are essential for initial containment of HSV‐2 replication, they may also underlie the three‐fold increased risk of HIV‐acquisition associated with HSV‐2 infection [201, 202]. Mucosal inflammation in the FGT is strongly associated with increased HIV acquisition risk, as it promotes the recruitment and activation of HIV target cells including CD4^+^ T cells, macrophages, and dendritic cells [157, 203, 204, 205]. Furthermore, pro‐inflammatory cytokine production by HSV‐2–infected ECs can impair epithelial barrier integrity, thereby facilitating viral entry and enhancing access to target cells [206]. Several AMPs are secreted when HSV‐2 infects the ECs of the FRT. Hazrati et al. [207] in their study involving both in‐vitro and in‐vivo models, showed that six α‐defensins:HNP1‐4, HD‐5, HD‐6 and the β‐defensin HBD‐3 could prevent the attachment and subsequent infection of host cells. They found that these AMPs could bind gB2, a glycoprotein on the surface of HSV‐2, or its receptor, heparan sulfate, preventing HSV‐2 from invading the host cells. HBD‐3 is the only AMP that can bind to both, gB2 and heparan sulfate at once, thus exhibiting the best anti‐HSV‐2 activity [207]. Wang et al. substituted the 21st glutamic acid of α‐defensin HD‐5 with arginine, strongly enhancing its binding affinity to HSV‐2 capsid protein gD, and in turn, its anti‐HSV‐2 activty [208]. Yasin et al. discovered that θ‐defensin retrocyclin 2 derived from human θ‐defensins pseudogene has a strong affinity for gB2 on HSV‐2 along with preventing the viru from infecting the host cell, in vitro [209]. Although SLPI has been shown to inhibit HSV‐2 infection in‐vitro, Fakioglu et al., showed that HSV‐2 significantly reduces the level of SLPI in human cervical ECs by either preventing its release, degrading it, or downregulating its expression [210]. Findings from an in‐vitro study by Drannik et al., show that Elafin and trappin‐2 decreased HSV‐2 binding, specifically interfering with its attachment to ECs of the reproductive tract and post viral entry, reducing viral replication by enhancing production of IFN‐ β and nuclear translocation of IRF3 [208, 209, 210, 211]. Ogawa et al. [212] showed that in‐vitro, ECs enhanced the production of soluble LL‐37 after HSV‐2 infection, but the exact mechanism still needs to be studied.

Given that clinical and experimental evidence indicates that women have a higher susceptibility to STIs than men, likely due to cyclic fluctuations in sex hormones, studies have shown that pre‐treatment of vaginal ECs with 10^−7^ M progesterone before HSV‐2 infection led to a significantly higher infection and viral replication when compared to estradiol [213, 214]. In contrast, treatment with 10^−9^ M 17β‐estradiol prior to infection resulted in markedly higher expression of cytokeratins, increased cell proliferation, and a significant reduction in viral shedding [213]. This estradiol concentration corresponds to levels observed during the early follicular phase, which follows menstruation and is associated with endometrial proliferation, while the progesterone concentrations used are equivalent to those found during the late proliferative stage to the secretory phase of the menstrual cycle (Figure 3) [215].

## 8 Functional and Transcriptional Regulation of FGT ECs by Sex Hormones and Hormonal Contraceptive

Although a substantial body of literature has examined the effects of endogenous sex hormones and hormonal contraceptives (HC) on FGT physiology and immune regulation within the reproductive tract, their direct effects on ECs remain comparatively underexplored (reviewed in [216]). Collectively, in vivo, in vitro, and animal studies demonstrate that endogenous sex hormones and the progestin‐based contraceptive medroxyprogesterone acetate (MPA) differentially regulate epithelial cell function in the FGT, with important implications for mucosal immunity and HIV‐1 susceptibility [111, 217, 218].

Several in vivo studies using mouse models have shown that removal of endogenous hormones through ovariectomy, or treatment with progesterone or HC—particularly depot medroxyprogesterone acetate (DMPA)—results in thinning of the vaginal epithelium, increasing susceptibility to HSV‐2 infection [213, 219]. However, the relevance of epithelial thinning to HIV‐1 acquisition remains unclear, and DMPA‐associated epithelial thinning has not been definitively demonstrated in the human FGT.

At the cellular level, multiple studies, including our own, show that estradiol enhances epithelial barrier integrity, whereas progesterone and DMPA do not confer similar protective effects. Genital epithelial cells (GECs) exhibit distinct transcriptional responses when exposed to physiological concentrations of estradiol (E2), progesterone (P4), or MPA. Although MPA was designed to mimic progesterone, microarray analyses of primary endometrial and vaginal ECs reveal that it induces a gene expression profile clearly distinct from that of P4. Specifically, MPA upregulates genes associated with inflammation and cholesterol/sterol biosynthesis—pathways linked to innate immune modulation and increased HIV‐1 susceptibility—while downregulating genes involved in cell division and cell–cell adhesion [217, 218]. Functionally, MPA, but not E2 or P4, increases epithelial permeability and inhibits cell cycle progression, findings consistent with compromised barrier integrity [218].

In contrast, endogenous hormones such as E2 and P4 modulate epithelial responses to acute HIV‐1 exposure by influencing the expression of genes involved in inflammation, interferon signaling, adhesion, and plasminogen activation [175]. Together, these findings indicate that while natural sex hormones shape epithelial immune responsiveness in a context‐dependent manner, MPA exerts distinct effects that may impair epithelial barrier function and potentially enhance susceptibility to HIV‐1 acquisition.

## 9 Conclusion

The interactions between sexually transmitted viruses and female genital ECs reflects a delicate balance between host defense mechanisms and viral strategies for persistence. Understanding the nuances of these interactions provides valuable insights into the early stages of transmission of sexually transmitted viruses and identifies potential targets for preventive and therapeutic interventions. Future research should focus on identifying and developing strategies to exploit epithelial innate immune responses to reinforce barrier integrity and enhance antiviral defenses. One promising avenue lies in the development of naturally derived therapeutic such as medicinal botanical products. For instance, studies from our lab have shown that curcumin, an active component of turmeric has anti‐inflammatory properties and can inactivate HIV‐1 (indirectly) and HSV‐2 directly [220]. Thus, administration of curcumin conjugated nanoparticles to the FGT can reduce inflammation in vivo and could be used as a potential therapeutic agent to reduce HIV acquisition in women [221]. In parallel, emerging molecular approaches offer unique avenues to modulate host‐virus interactions. MicroRNAs, small non‐coding single stranded RNA molecules regulate post‐transcriptional gene expression and influence disease outcomes [222]. There are several host microRNAs that regulate HIV infection at different stages‐from entry to viral release. Although more clinical research is required to fully evaluate the potential of microRNAs to treat HIV‐1 infection and associated complications, targeting these microRNAs with mimics and inhibitors is a possible therapeutic approach against HIV‐1 [223]. Collectively, integrating barrier‐enhancing and anti‐inflammatory therapeutics with precision molecular interventions may provide a multifaceted approach to reducing sexually transmitted viral infections in women.

## Funding

This study is funded by a research grant # 159229 from CIHR to Dr. Charu Kaushic.

## Ethics Statement

The authors confirm that the ethical policies of the journal, as noted on the journal's author guidelines page, have been adhered to. No ethical approval was required as this is a review article with no original research data.

## Conflicts of Interest

The authors declare that the research was conducted in the absence of any commercial or financial relationships that could be construed as a potential conflict of interest.

## Data Availability

Data sharing not applicable to this article as no datasets were generated or analyzed during the current study.
